# Dg-Dys-Syn1 signaling in *Drosophila* regulates the microRNA profile

**DOI:** 10.1186/1471-2121-13-26

**Published:** 2012-10-29

**Authors:** April K Marrone, Evgeniia V Edeleva, Mariya M Kucherenko, Nai-Hua Hsiao, Halyna R Shcherbata

**Affiliations:** 1 Max Planck Research Group of Gene Expression and Signaling, Max Planck Institute for biophysical chemistry, Am Fassberg 11, Goettingen 37077, Germany

**Keywords:** Dystrophin, Dystroglycan, Syntrophin, microRNAs, Neuromuscular disorders

## Abstract

**Background:**

The Dystrophin Glycoprotein Complex (DGC) is at the center of significant inheritable diseases, such as muscular dystrophies that can be fatal and impair neuronal function in addition to muscle degeneration. Recent evidence has shown that it can control cellular homeostasis and work via Dystrophin signaling to regulate microRNA gene expression which implies that disease phenotypes hide an entourage of regulatory and homeostatic anomalies. Uncovering these hidden processes could shed new light on the importance of proper DGC function for an organism’s overall welfare and bring forth new ideas for treatments.

**Results:**

To better understand a role for the DGC in these processes, we used the genetically advantageous *Drosophila* muscular dystrophy model to conduct a whole animal microarray screen. Since we have recently found that dystrophic symptoms can be caused by stress even in wild type animals and are enhanced in mutants, we screened stressed animals for microRNA misregulation as well. We were able to define microRNAs misregulated due to stress and/or dystrophy. Our results support the hypothesis that there is a Dystrophin and Dystroglycan dependent circuitry of processes linking stress response, dystrophic conditions and cellular signaling and that microRNAs play an important role in this network. Verification of a subset of our results was conducted via q-PCR and revealed that miR-956, miR-980 and miR-252 are regulated via a Dystroglycan-Dystrophin-Syntrophin dependent pathway.

**Conclusions:**

The results presented in this study support the hypothesis that there is a Dystrophin and Dystroglycan dependent circuitry of processes that includes regulation of microRNAs. Dystrophin signaling has already been found to occur in mammalian musculature; however, our data reveals that this regulation is evolutionarily conserved and also present in at least neuronal tissues. Our data imply that Dystroglycan-Dystrophin-Syntrophin signaling through control of multiple microRNAs is involved in highly managed regulation of gene expression required to adapt cellular homeostasis that is compromised under stress and dystrophic conditions.

## Background

Muscular dystrophies (MDs) are inherited, sometimes fatal diseases associated with progressive muscle wasting and brain defects [1,2]. In addition to these well-known symptoms, reports have also shown that MDs can cause problematic metabolism, oxidative and energetic stress, and overproliferation of muscle satellite cells [3-7]. MDs can be genetically linked to mutations in the Dystrophin Glycoprotein Complex (DGC) which in mammals generally consists of the very large dystrophin protein, dystroglycans α and β, sarcoglycans α, β, γ and δ, sarcospan and numerous other proteins including syntrophins α1, β1, γ1 and γ2 [8].

In addition to providing structural support to muscles, the DGC has also been shown to be involved in general signaling pathways. For example, dystroglycan binding to laminin and dystrophin binding to syntrophins is necessary for G-protein activation of PI3K/Akt and Syntrophin-Grb2-Sos1-Rac1-Pak1-JNK signaling [9,10]. Dystrophin has also recently been linked to microRNA (miRNA) regulation where dystrophin deficiency in mice and humans causes altered miRNA profiles (reviewed in [11]). In particular, the DGC via dystrophin-syntrophin-neuronal Nitric Oxide Synthase signaling can regulate the expression of miRNAs via histone modification [12]. Accordingly, patients with the severe Duchenne MD resulting from the loss of dystrophin have altered gene transcript levels in muscles correlating with misexpressed miRNAs [13], and the dystrophin deficient nervous system has distorted gene expression in mice [14]. Due to general signaling and the control over expression of miRNAs and downstream gene transcripts, it is now believed that there are numerous cellular processes that are impacted by mutations in DGC components.

The DGC is conserved and most of the involved proteins have homologues in *Drosophila*[15] that have been shown to be expressed in muscles and the nervous system [16]. We specifically study *Dystrophin* (*Dys*) and *Dystroglycan* (*Dg*) mutants which we have used to study the role of these proteins in muscle and the nervous system. As in mammals, loss of Dys and Dg results in age related muscle wasting, and several proteins genetically interact with Dys and/or Dg causing muscle degeneration [17,18]. Most importantly, stress can cause muscle degeneration even in wild type animals and can accelerate it in dystrophic mutants. Additionally, Dg is involved in maintaining muscle cell homeostasis under energetic stress [18]. In the *Drosophila* nervous system, Dys and Dg have a role in the developing visual system and can impact the ability of axons to migrate properly [19]. Due to these findings, we hypothesize that some shared processes are perturbed under stress and dystrophic conditions which can explain why dystrophic phenotypes can be recovered simply by inducing stress.

Our aim here is to improve our understanding of how stress and dystrophy are related to one another. miRNAs have been implicated in stress response before [20,21] and, as previously noted, mutations in Dys can cause miRNA misregulation [12,13,22]. Therefore, we conducted a miRNA microarray screen in *Dys* and *Dg* loss of function mutants and in stressed *wild type* (*wt*) and mutant animals. Here we used whole animals to screen for misregulated miRNAs to find altered levels in tissues other than muscle, including the nervous system. We found that similarities are shared in miRNA profiles under stress and dystrophic conditions, that loss of Dg and/or Dys can alter miRNA levels and that signaling via Syntrophin-like 1 (Syn1) controls the expression levels of miR-252, miR-956 and miR-980. The regulation of miR-252 and miR-980 is not restricted to the musculature, but occurs also in the nervous system. Even though it is not common for miRNAs to be evolutionarily conserved, the processes that they regulate are. Thus, our results help gain a better understanding of how organismal homeostasis is regulated by the DGC and associated miRNAs.

## Results

### Stress and dystrophy alter the miRNA profile in Drosophila

**Figure 1:**
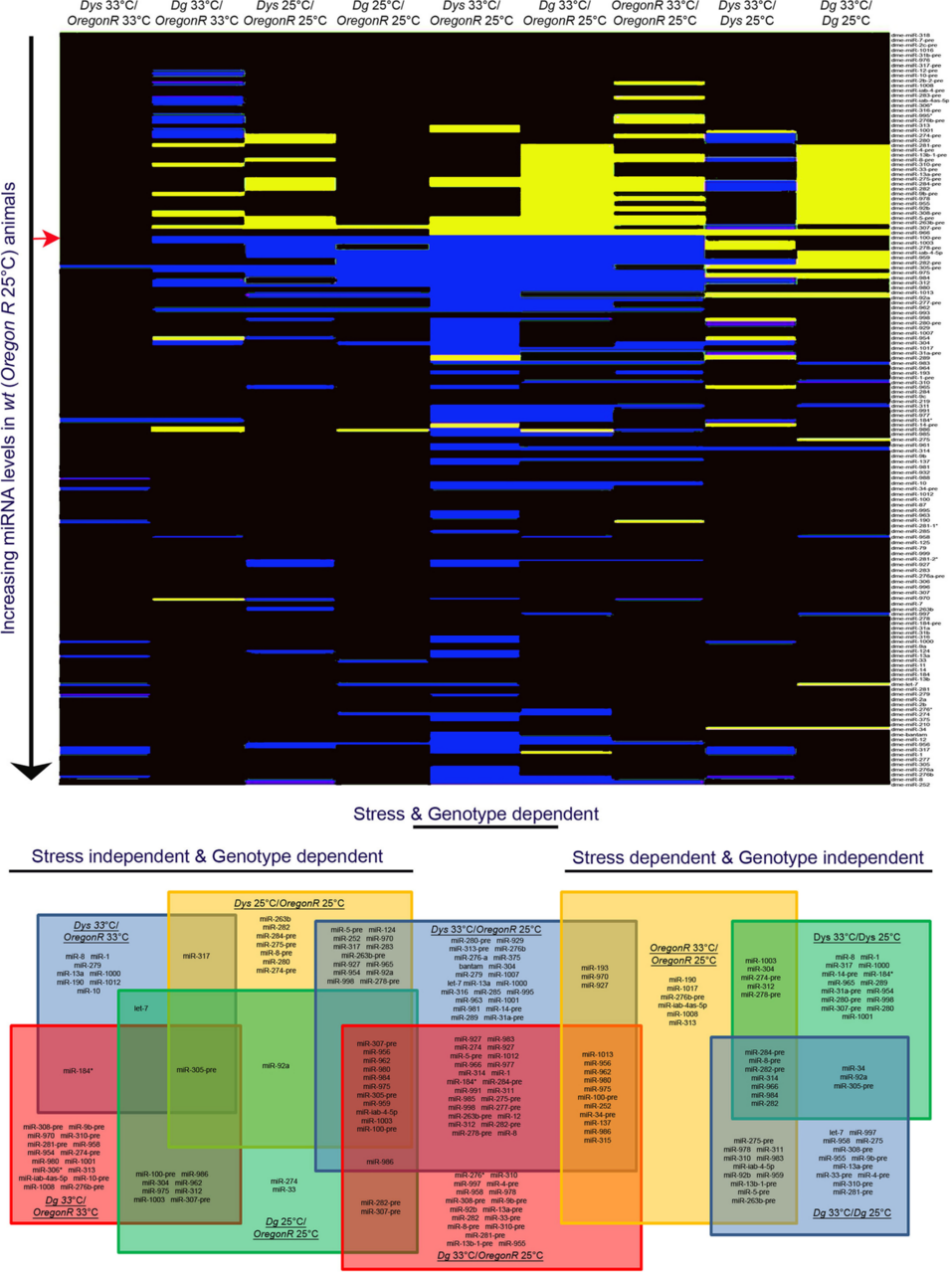
**Stress and Loss of Dys and/or Dg alters miRNA expression levels. a**) A heatmap of microarray screen data shows that several miRNAs are misregulated when animals are missing Dys and/or Dg. High temperature stress can also induce a change in miRNA expression levels. Genotypes are written across the top where the bottom genotype is the normalization factor for each column. Black indicates no change, yellow upregulation and blue downregulation of the miRNAs listed in the column to the right. Only miRNAs that had a signal above the threshold under at least one of the tested conditions were mapped. miRNAs are listed in the order of increasing levels measured in *Oregon R *animals where miRNAs below the red arrow were found to be expressed in *Oregon R *adult males at 25°C. miRNAs altered in each comparison can be grouped into being independent of stress but dependent on genotype, dependent on stress and genotype, or dependent upon stress regardless of the genotype. Misregulated miRNAs fitting into these categories are represented in the bottom portion of the figure in the Venn diagram.

We used 3–5 day old adult male flies to profile the levels of miRNAs via microarray in *wt* (*Oregon R*), *Dys* (*DysDf/DysDf*) and *Dg* (*Dg*^*O86*^*/Dg*^*O55*^) mutants both under normal and stress conditions. High temperature is a good stressor to recover dystrophic symptoms in wild type animals, and accelerates muscle degeneration and alters metabolic function in mutants [18,23]. Therefore, to stress animals for this study, they were kept for 3 days at 33°C. Young animals were used because they do not have muscle wasting yet, and we did not want to detect altered miRNA levels due to degenerative processes, but rather altered cellular processes. Based on miRNAs annotated in miRBase release 16 [24], 146 miRNAs, 76 pre-miRNAs and 6 anti-sense miRNAs were tested. Of these, 110 mature miRNAs were found to give a signal above the background threshold in at least one of the genotypes/conditions tested (Figure 1, Additional file 1). Nine comparisons were possible based on having three genotypes in two conditions each. The results could then be categorized by miRNAs that were altered dependent upon genotype only, those altered dependent upon stress and genotype, and those that were altered dependent upon stress only. Based on this large set of data allowing for many comparisons, we divided the data into functional sub-categories. Though of possible future interest, pre- and anti-sense miRNAs were not considered further.

### miRNA expression profiles link stress, dystrophy and DGC signaling

To assess the microarray derived miRNA levels in this study, we make several assumptions allowing us to categorize misregulated miRNAs based on presumed biological roles. First, we contend that there are common biological problems that are shared in dystrophic and stress conditions that can explain why we can recover dystrophic phenotypes by inducing stress. Accordingly, the downregulation of a miRNA in a mutant at an ambient, non-stressful temperature could also be downregulated in the *wt* animal at a stressful temperature. Therefore, the comparison of a stressed mutant and a stressed *wt* animal may show no difference in a miRNA level due to a shared biological problem. Furthermore, if a mutation causes a miRNA to be misregulated at the ambient temperature then that same miRNA should be misregulated similarly in the mutant at 33°C relative to *wt* at the ambient temperature. Based on this logic, we assigned miRNAs into functional categories.

**Figure 2:**
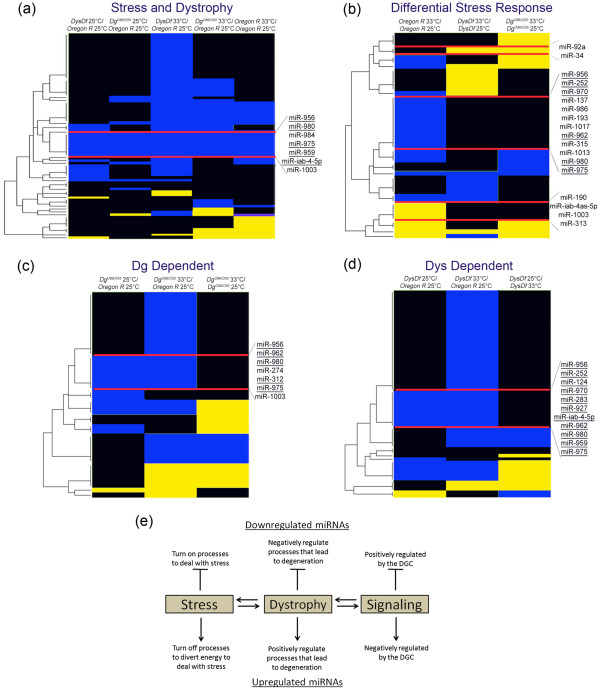
**Altered miRNAs are shared among functionally relevant categories. **(**a**-**d**) Heatmaps of altered miRNAs grouped into functionally relevant categories: only miRNAs that were altered in the indicated comparisons were included in generating the heatmaps per grouping. Underlined miRNAs were further tested for microarray agreement via q-PCR. (**a**) Combination of both hyperthermic stress and dystrophy, (**b**) differential response based on stress in mutants, (**c**) dependent on the loss of Dg and (**d**) dependent on the loss of Dys. (**e**) Stress, dystrophy and Dg/Dys signaling all can cause altered levels of miRNAs that are common among each category showing a functional link in these processes.

The first category consists of miRNAs that are altered based on stress and dystrophy (Figure 2a). These miRNAs are downregulated due to mutations in both *Dys* and *Dg* at both temperatures relative to *wt* animals kept at 25°C. Also, stressed *wt* animals have the same miRNAs downregulated. Dystrophic miRNAs revealed are miR-956, miR-980, miR-984, miR-975, miR-959, miR-iab-4-5p and miR-1003.

The next category is DGC differential stress response miRNAs. When we use hyperthermia to induce stress we can see that there is a miRNA response (Figure 2b). Some miRNA levels decrease, and a subset of miRNAs that are usually not expressed at 25°C are ‘turned on’. These could be protective miRNAs that are expressed to regulate the negative effects of stress. Stressed mutants also respond, but since dystrophic muscles are already compromised (and as a consequence, they are less adaptive and more sensitive to energetic stress and changes in the ambient temperature) miRNA levels do not change in the same way as in *wt* animals. There can be two sub-categories delineated; miRNAs that do not change normally under stress, but do in dystrophic mutants (miR-92a and miR-34) and miRNAs that change as a normal response, but do not in *Dys* and *Dg* mutants (miR-956, miR-252, miR-970, miR-137, miR-986, miR-193, miR-1017, miR-962, miR-315, miR-1013, miR-980, miR-975, miR-190, miR-iab-4as-5p, miR-1003 and miR-313).

Since there are similarities in stress and dystrophy miRNA regulation, and shared phenotypes in mutant dystrophic and stressed animals [18], we are also interested in how the dystrophic genotypes can modify miRNA levels. To this extent, we can see that loss of Dg and Dys leads to altered miRNA profiles (Figure 2c,d). To fit into one of these clusters the miRNA must be downregulated due to the mutation (*Dys* or *Dg*) at both 25°C and 33°C relative to the *wt* control at the ambient temperature. In addition, to eliminate hyperthermia response miRNAs, levels should not change in the mutant at different temperatures. These criteria return miRNAs that are misregulated due to mutations alone based on the assumptions outlined above. We found that potential Dg-regulated miRNAs are miR-956, miR-962, miR-980, miR-274, miR-312, miR-975, and miR-1003. Potential Dys-regulated miRNAs are miR-956, miR-252, miR-980, miR-124, miR-970, miR-283, miR-927, miR-iab-4-5p, miR-962, miR-959, and miR-975. Worth noting, is that of all of the 110 mature miRNAs found by the microarray to be expressed in the adult male, there is great redundancy in those misregulated between the four functional groups. In total 65% of the isolated microarray identified miRNAs are common to more than one of the defined functional groups (28 out of 43). This implies that stress, dystrophy and Dys/Dg signaling are interconnected in the biology of the entire organism (Figure 2e). Next, we verified data derived from the microarray screen.

### Verification of microarray data points to miRNA regulation by stress

We tested levels of 15 miRNAs in *wt* and mutant animals (11 from the functional groups and 4 as negative controls) in both stress and non-stress conditions using TaqMan microRNA quantitative PCR (q-PCR) assays resulting in 135 comparisons. We were particularly interested in Dys signaling miRNA regulation, thus we tested the “Dys Dependent” cluster from Figure 2d: miR-956, miR-252, miR-124, miR-970, miR-283, miR-927, miR-iab-4-5p, miR-962, miR-980, miR-959, and miR-975 (Additional file 1).

**Figure 3:**
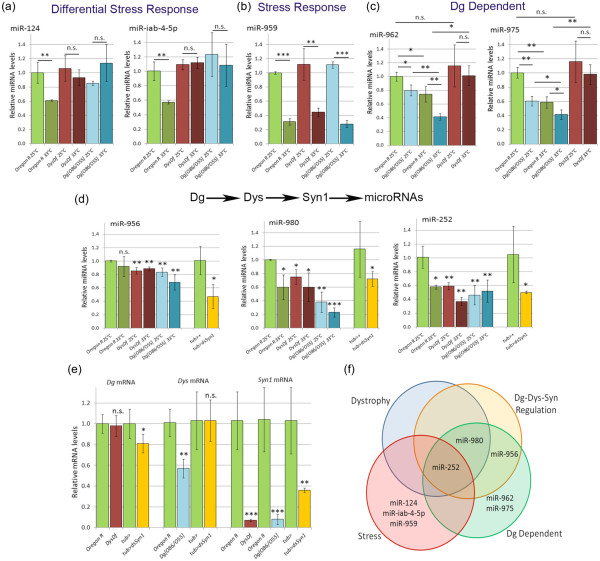
**q-PCR of microarray data reveals miRNAs involved in stress response and associated with Dys and Dg signaling. **q-PCR verification of microarray screen reveals miRNAs that fall into each functional category. (**a**) miR-124 and miR-iab-4-5p levels do not go down in *Dys *and *Dg *mutants when high temperature stress is applied. (**b**) miR-959 was found to be a general temperature stress response miRNA. (**c**) miR-962 and miR-975 have reduced levels in response to loss of Dg alone. (**d**) miR-956, miR-252 and miR-980 all are downregulated in response to loss of Dg, Dys and Syn1. (**e**) Relative levels of *Dg *mRNA in *Dys *and *dsSyn1 *mutants, *Dys* mRNA levels in *Dg *and *dsSyn1 *mutants and relative levels of *Syn1* mRNA in *Dys, Dg *and *dsSyn1 *mutants. Each comparison is made to the closest control genotype (*Oregon R *or *tub-Gal4>+*) to the left of the test genotype. Statistics were done using a one-tailed Student’s t-test: *P ≤ 0.05, **P ≤ 0.01, ***P ≤ 0.001, n.s – not significant. (**f**) Venn diagram shows overlap among verified miRNAs and the relevant functional groups.

Based on the verification we were explicitly able to assign functionality to the subset of tested miRNAs. First miR-124 and miR-iab-4-5p are downregulated in *wt* animals in response to hyperthermic stress, but their levels are not altered appropriately in *Dys* and *Dg* mutants. Thus, they are classified as Dys/Dg dependent stress response miRNAs (Figure 3a). We also found that miR-959 is a general high temperature stress response miRNA (Figure 3b). The level of miR-959 is decreased at 33°C in all three genotypes, and is not affected by the mutations. miR-962 and miR-975 levels are decreased due to the absence of Dg at ambient temperature and these miRNAs are downregulated at the higher temperature in both *wt* and *Dg* mutants (Figure 3c). However, at 33°C both of these miRNA’s levels remain at the ambient temperature levels in *Dys* mutants. Interestingly, appropriate levels of miR-252, miR-956 and miR-980 depend on both Dg and Dys (Figure 3d).

Of the 135 comparisons made using q-PCR, 79 (58.5%) agreed with the microarray data. Recently a RT q-PCR based array has become available that uses the same stem-loop RT technology as the Taqman assays used in our verification studies [25]. Subsequently, a study was undertaken that specifically compared the q-PCR array technology to the standard microarray technology used here [26]. Surprisingly, this group reported that the two methods only had a correlation of −0.443 and most interestingly, the microarray found 13% of miRNAs to be differentially expressed compared to only 1% of tested miRNAs by the q-PCR array. The finding of that study supports our work here in regards to the low percentage of agreement between the microarray data and that verified via q-PCR and emphasizes the importance of proper verification in these types of studies in general.

### Verification of microarray data points to miRNA regulation by the Dg-Dys-Syn1 pathway

It has been reported before that miRNAs can be regulated via Dys-Syn-nNOS signaling in mammals [12], thus we wanted to know if the three miRNAs that are downregulated in *Dg* and *Dys* mutants are also affected by reduction of Syntrophin. There are two Syntrophin genes in *Drosophila*, Syntrophin-like 1 (Syn1) is homologous to α1/β1/β2-syntrophins and Syntrophin-like 2 (Syn2) is homologous to γ1/γ2-syntrophins. We chose to examine the effect of *Syn1* reduction via *RNAi* (*dsSyn1*) on the levels of miR-252, miR-956 and miR-980, since in mammals it was found that α1/β1/β2-syntrophins bind to nNOS [27]. To downregulate Syn1, we used a *Syn1 RNAi* construct driven by a ubiquitous *Gal4* driver (*tub-Gal4/dsSyn1*) which resulted in a ~65% reduction of *Syn1* expression (Figure 3d, Additional file 1). We also found that in addition to the loss of *Dys* and *Dg*, reduction of *Syn1* caused a decrease in all three miRNA’s levels (Figure 3d, Additional file 1). These data suggest that there is a similar regulatory pathway in *Drosophila* for the management of miRNA levels by the Dg-Dys-Syn1 pathway.

Importantly, Dg, Dys, and Syn1 expression levels could be mutually dependent; the loss of one of these proteins could affect the levels of the others. To determine if this is the case we measured the levels of *Dys* mRNA in *Dg* and *dsSyn1* mutants, *Dg* mRNA levels in *Dys* and *dsSyn1* mutants, and *Syn1* mRNA levels in both *Dys* and *Dg* mutants. We found that the loss of Dys did not affect the levels of *Dg* mRNA, but the loss of Dg does lower the amount of *Dys* mRNA (Figure 3e, Additional file 1). Additionally, loss of Dys or Dg causes a reduction in *Syn1* mRNA levels. *Dg* levels are slightly decreased (~20%) in *dsSyn1* mutants, but *Dys* levels are not altered. Overall our results reveal the existence of Dg-Dys-Syn1 signaling, which involves miRNAs and regulates biological processes in dystrophic conditions (Figure 3f).

### Putative mRNA targets of Dg-Dys-Syn1 regulated miRNAs are involved in general cellular processes, the nervous system, and muscle development and maintenance

miRNAs provide a strategy for gene regulation mainly by interacting with the 3^′^untranslated regions (UTRs) of target transcripts [28-31]. To determine what biological significance the regulation of the three identified DGC miRNAs could have, we determined their putative targets and their possible biological roles based on these targets. We first derived lists of potential conserved (among *Drosophila* species) genetic targets using the TargetScan database [32] and compared them to predicted targets from microRNA.org [33] for consensus. The individual lists of target genes per miRNA were then analyzed using the Generic Gene Ontology (GO) Term Finder Tool [34].

**Figure 4:**
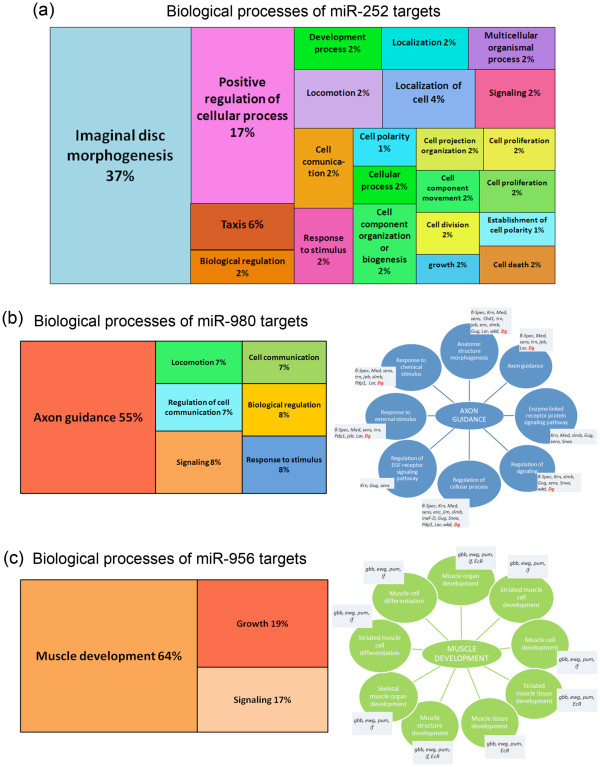
**Dg-Dys-Syn1 regulates levels of miRNAs with diverse mRNA targets. **Gene ontology predictions of putative (**a**) miR-252, (**b**) miR-980 and (**c**) miR-956 target genes. (**a**) The majority of miR-252 targets are involved in imaginal disc morphogenesis next to regulation of cellular process. (**b**) More than half of the putative miR-980 targets are involved in processes essential for axon guidance, whereas the majority of miR-956 targets are involved in muscle development. Percentages are based on the number of target genes involved in the shown process where predictions are based on a p ≤ 0.01.

miR-252 is the most highly expressed miRNA in the adult fly, and it has 206 putative targets that are involved in a large range of biological processes which agree among the two databases. The most notable categories of miR-252 predicted targets are imaginal disc morphogenesis and regulation of cellular processes (Figure 4a, Additional file 1). Due to the large number of targets and GO terms, it is difficult to assign one specific function for miR-252.

After careful analysis, miR-980 showed 24 putative target genes (Additional file 1) and the majority of them are involved in axon guidance (Figure 4b). Interestingly, *Dg* is a putative target identified by both databases involved in most of the axon guidance processes, but it is not a conserved target among *Drosophila* species. Due to this, Dg was not included in the GO analysis.

We additionally found miR-956 to have 19 putative target genes (Additional file 1), and the majority of these are involved in muscle development (Figure 4c). Because miR-956, miR-980 and miR-252 are predicted to be involved in varied processes and are regulated via the Dg-Dys-Syn1 pathway, we hypothesize that this signaling plays a role in general cellular processes of the nervous system and muscle via miRNA regulation. Ongoing efforts should shed light on this proposal in the future.

### Dg-Dys-Syn1 regulated miRNAs are expressed in the nervous system and musculature

To determine when and where the Dg-Dys-Syn1 regulated miRNAs are expressed, we performed reverse transcription quantitative PCR (RT q-PCR) for these miRNAs from RNAs extracted at different developmental time points and from individual adult body parts. All levels were normalized to those of the whole adult male fly. We additionally analyzed larval brains and muscles using Locked Nucleic Acid (LNA) *in situ* hybridization with probes targeting miR-956, miR-980 and miR-252.

**Figure 5:**
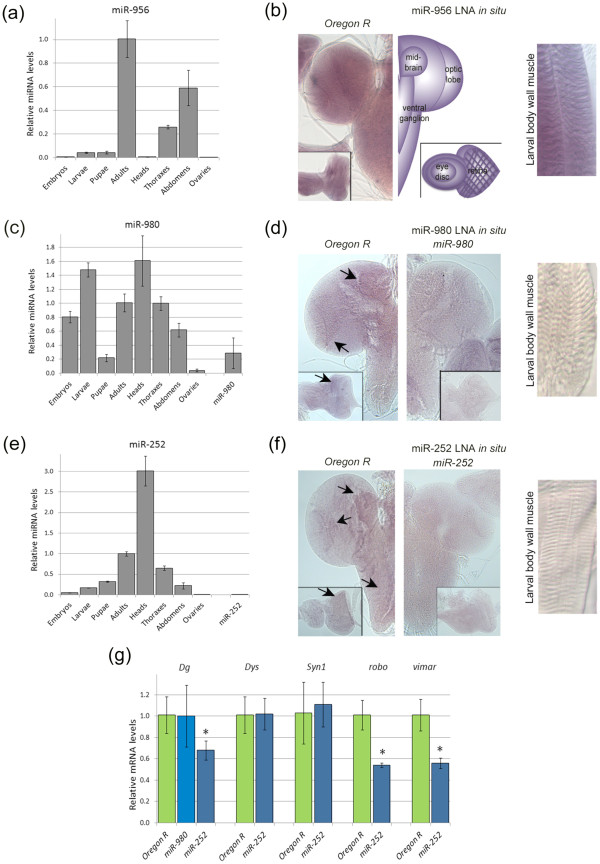
**Dg-Dys-Syn1 regulates levels of miRNAs found in the nervous system and musculature. **q-PCR analysis revealed the expression levels during all life stages and in adult body parts for (**a**) miR-956 (**c**) miR-980 and (**e**) miR-252. All levels have been normalized to those found in whole adult male flies. (**b**,**d** and **f**) LNA *in situ *hybridization: (**b**) miR-956 is present in the 3^rd ^instar larval brain, eye discs and body wall muscles. (**d**) miR-980 is present in the 3^rd ^instar larval brain in axons of photoreceptor cells in the optic lobe, in the eye disc, and in the midbrain. Note the reduced expression levels in *miR-980 *hypomorphs. miR-980 expression was not detected in the larval body wall muscles. (**f**) LNA *in situ *hybridization shows neuronal miR-252 in the optic lobe, eye disc, midbrain and ventral ganglion. Expression is not detected in the *miR-252 *mutant. miR-252 expression was not detected in larval body wall muscles. (**g**) RT q-PCR analysis of selected predicted target genes *Dg*, *Dys*, *Syn1*, *robo *and *vimar *in *miR-252 *amorphs and *Dg *in *miR-980 *hypomorphs. Three independent RNA extractions were conducted per genotype. Statistics were done using a two-tailed Student’s t-test: *P ≤ 0.05, **P ≤ 0.01, ***P ≤ 0.001.

miR-956 is expressed at a very low level in the adult head and is primarily detected in the adult thorax and abdomen (Figure 5a). A reasonable hypothesis based on these data and the GO of predicted target genes is that miR-956 is expressed in the adult animal in the muscles of the thorax and abdomen to repress muscle development genes. The relatively low level of miR-956 during embryonic, larval and pupal stages when muscles actively develop and grow supports this hypothesis. *In situ* hybridization revealed a strong ubiquitous signal in the larval brain and muscles (Figure 5b). Though expression in the muscle tissue was expected, expression in the brain was not. It will be of future interest to explore this finding which will be dependent upon the generation of an appropriate *miR-956* mutant.

miR-980 has mostly nervous system predicted targets and is expressed during embryogenesis and the larval stage, but a decrease in levels is seen in pupae (Figure 5c). This could be indicative of a role in repressing target genes that are critical for sequences of temporal processes such as nervous system remodeling. Once metamorphosis is complete, miR-980 levels are elevated again, and the highest relative level is noted in the head. Analysis of miR-980 expression shows that it is expressed in the developing eye, the axons of photoreceptor neurons and in the midbrain (Figure 5d). The Flybase annotated allele *P[GawB]CG3777*^*NP3544*^ disrupts *miR-980*, but does not disturb the mature sequence. The result is a hypomorphic homozygous viable mutant that reduces miR-980 levels by ~70% analyzed via RT q-PCR at the ambient temperature (Figure 5c). In accord with the reduced miR-980 levels, *miR-980* mutants have a reduced, but not abolished LNA hybridization signal (Figure 5d) which supports the specificity of miR-980 expression detected by the LNA probe. Interestingly, no expression was detected in larval body wall muscles (Figure 5d). miR-980 expression in the adult body could be from the peripheral nervous system including the thoracic ganglion, a large structure located in the thorax.

miR-252 levels gradually increase throughout development, and the expression is highest in the adult (Figure 5e). Since miR-252 is predicted to downregulate genes involved in morphogenesis perhaps its levels are decreased to allow for progression of such processes during developmental stages. Of the individual body parts, miR-252 is seen to be elevated threefold in the head relative to the whole body. An expression pattern similar to the expression of miR-980 is seen for miR-252 (Figure 5f). Expression is evident in the eye disc and optic lobe. Additionally, a signal is seen in the midbrain and the ventral ganglion (Figure 5f). The Flybase annotated allele *PBac[SAstopDsRed]LL04028* disrupts the mature sequence of *miR-252* resulting in a homozygous viable null mutant analyzed via RT q-PCR (Figure 5e). In agreement with this being a loss of function mutant, *miR-252* mutants do not show any apparent expression pattern. Even though our data show that miR-252 levels are changed due to muscular dystrophy, expression was not detected in larval body wall muscles. The expression profiling and detection of miR-956, miR-980 and miR-252 supports our hypothesis that Dg-Dys-Syn1 signaling is part of an organism’s overall development and homeostasis.

### miR-252 can regulate levels of DGC interacting components

Previously large scale screening found *Dys* and/or *Dg* genetic interactors using an easily detectable wing vein phenotype [35] and identified numerous potential genes. Further screening was then conducted to determine which of those interactors also affected muscle degeneration [18]. We have found in this study three miRNAs that are regulated by both Dys and Dg. Therefore, we examined 3^′^UTR sequences of the previously identified genetic interactors to see if any of them are possible targets of these miRNAs. We found that miR-980, miR-956 and to a greater extent, miR-252 can potentially target a portion of them (Table 1). Of the 37 genes that were found in the primary screen to interact with *Dys* and/or *Dg*, 17 of them (45.9%) can potentially be targeted by the miRNAs identified to be involved in the Dg-Dys-Syn1 pathway here. Interestingly, miR-980 can potentially target *Dg* while *Dg*, *Dys* and *Syn1* are putative miR-252 targets. In addition to these main DGC components, miR-252 can potentially target *robo* (roundabout) and *vimar* (visceral mesodermal armadillo-repeats)*,* which were found to interact with *Dys* and/or *Dg* in the muscle degeneration process. Calmodulin (*Cam*) is also a non-conserved possible target of miR-252 that was found to not only interact with *Dys* in wing vein formation and muscle degeneration, but to genetically cooperate with both *Dys* and *Dg* in axon path finding and photoreceptor differentiation [19].

**Table 1:** DGC interactors are putative Dg-Dys-Syn1 regulated miRNA targets

**Gene name**	**Symbol**	**Function**	Targeting miRNA*	**Interaction**	
				**Dg**	**Dys**
Dystrophin	*Dys*	Main DGC component	miR-252	w, m	-
Dystroglycan	*Dg*	Extra- intracellular signaling	miR-252	-	w, m
			miR-980		
Syntrophin-like 1	*Syn1*	structural constituent of muscle; cytoskeletal protein binding	miR-252	nt	nt
Calmodulin	*Cam*	Ca^2+^ dependent pathways regulation	miR-252*	-	w, m
Kinesin heavy chain	*Khc*	microtubule motor activity	miR-252*	-	w
Sema-2a	*Sema-2a*	protein binding	miR-252	w	w
Sema-1a	*Sema-1a*	protein binding	miR-252*	w	w
frazzled	*fra*	netrin receptor	miR-252	-	w
roundabout	*robo*	Heparin/protein binding	miR-252	w, m	w, m
Syndecan	*Sdc*	transmembrane receptor	miR-980*	-	w
starry night	*stan*	receptor signaling	miR-252*	w	w
Delta	*Dl*	Notch binding	miR-252	w	w
			miR-980*		
Daughters against dpp	*Dad*	TGF-β receptor	miR-252*	w	w
thickveins	*tkv*	TGF-β receptor	miR-252*	-	w
homeodomain interacting protein kinase	*hipk*	β-catenin binding; transcription factor binding; protein serine/threonine kinase	miR-980*	-	w
			miR-956*		
kismet	*kis*	ATP-dependent helicase	miR-252	w	w
CG4496	*CG4496*	zinc ion binding; nucleic acid binding	miR-252*	-	w
Plenty of SH3s	*POSH*	ubiquitin-protein ligase	miR-252	w	w
visceral mesodermal armadillo-repeats	*vimar*	Ral GTPase binding	miR-252	w, m	w
uninflatable	*uif*	Notch binding	miR-980*	-	w

* non-conserved target site among *Drosophila *species.

w - interaction published to occur in wing development [35].

m - interaction published to occur in muscle maintenance [18].

nt = not tested.

Mature miRNAs are incorporated into the RNA-induced silencing complex (RISC), where this complex targets mRNAs by annealing to the 3^′^UTR and promoting either mRNA degradation or translational inhibition [36]. Since it has been found that the most robust mRNA targets of miRNAs can be detected by variation in mRNA levels [37,38], we tested the putative DGC component and conserved muscle targets via RT q-PCR in *miR-980* and *miR-252* mutants. If the mRNAs tested are direct targets of the miRNAs, then we would expect to see an increase in the mRNA levels in the mutants. *Dg* mRNA levels were not altered in hypomorphic *miR-980* mutants (Figure 5g), but miR-980 levels are not completely abolished. Interestingly, the tested DGC components and interactors predicted to be miR-252 targets cannot be directly downregulated by this miRNA (Figure 5g); however, loss of miR-252 negatively affects mRNA levels of *Dg*, *robo* and *vimar*. These results suggest that miR-252 is involved in the regulatory circuitry maintaining DGC associated protein levels.

Robo has been studied extensively in the past and is part of the Slit-Robo pathway involved in axon guidance and brain compartmentalization [39,40]. However, Vimar has been less studied: the founding report presented evidence that it is expressed in the embryonic midgut and hindgut visceral mesoderm, as well as in the CNS and PNS [41]. Another screen found Vimar to be required for proper mitochondrial function [42]. The regulation of these diverse proteins suggests that the overall cellular processes that are controlled by the Dg-Dys-Syn1-miR-252 pathway are diverse and related to the dystrophic symptoms found in our previous works.

### miR-980 regulates stress response in *Drosophila*

**Figure 6:**
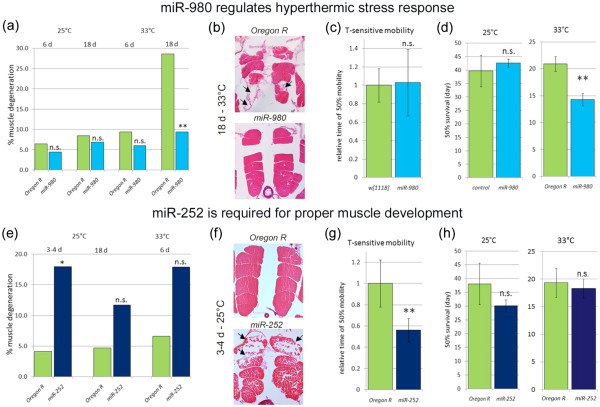
**miR-980 is involved in stress response and miR-252 is required for muscle development. **(**a**) Muscle degeneration assayed in *wt *and *miR-980* mutants kept for 6 and 18 days at 25°C and 33°C. (**b**) Exemplary histological sections of control and *miR-980 *muscles after 18 d at 33°C. (**c**) High temperature (~38°C) mobility assay revealed no difference between *wt *and *miR-980 *mutants. (**d**) When housed at 25°C, *miR-980 *mutants did not have a shortened lifespan compared to control *w*^*1118*^/*OregonR*, but at 33°C their lifespan was significantly lower than in wild type animals. (**e**) Muscle degeneration assayed in young, old and stressed *wt *and *miR-252 *mutants. (**f**) Exemplary histological sections of control and *miR-252 *muscles from 3–4 d old flies. (**g**) High temperature (~38°C) mobility assay revealed that *miR-252 *mutants have mobility defects under hyperthermic stress. (**h**) When housed at 25°C and 33°C, *miR-252 *mutants did not have a shortened lifespan. Muscle degeneration was compared using the Chi-squared statistic where statistical significance of the other assays was done with the Student’s two-tailed t-test: *P ≤ 0.05, **P ≤ 0.01, ***P ≤ 0.001.

As can be seen in Figure 3d, miR-980 levels were also decreased due to hyperthermic stress. Therefore, we next wanted to determine if miR-980 is involved in stress biology. We first assayed muscle degeneration of *miR-980* hypomorphic mutants and control animals housed at both 25°C and 33°C. As would be expected based on our prior report [18], the control animals had muscle degeneration caused by the high temperature stress after eighteen days relative to those kept at 25°C (28.6% vs. 8.43%, Table 2, Figure 6a). Interestingly, the reduction of miR-980 preserved the muscles after the same time period at 33°C relative to 25°C (Table 2, Figure 6a,b).

**Table 2:** **Muscular dystrophy-related phenotypes in *miR-980 *mutants**

**Genotype**	**Longevity**		**Temperature-sensitive mobility**	**Muscle degeneration**			
	***50% survival (day)***		***Relative time of 50% mobility***	***6 days***	***18 days***	***6 days***	***18 days***
	**25°C**	**33°C**	**38±1°C**	**25°C**		**33°C**	
*Control*	39.7±5.86	21.0±1.3	1.00±0.18	6.45%	8.42%	9.35%	28.63%
	(n=90)	(n=90)	(n=70)	(n=217)	(n=178)	(n=246)	(n=234)
*miR-980*	42.7±1.53	14.3±1.2	1.03±0.36	4.39%	6.85%	5.99%	9.42%
	(n=90)	(n=150)	(n=100)	(n=182)	(n=219)	(n=267)	(n=329)

In a further effort to see how *miR-980* mutants respond to stress, we utilized a hyperthermic mobility assay [18]. Animals are placed in a vial that is pre-heated to ~38°C and are scored every 30 s for mobility. Animals that have muscle and/or neuromuscular problems may start to seize and/or become paralyzed. We did not observe *miR-980* mutants to have mobility defects in this assay (Figure 6c). However, animals that were housed in 33°C did lose the ability to move at approximately fourteen days while the *wt* flies still performed normally. The timing of this inability to move coincided with the onset when *miR-980* mutants started to die (Table 2).

Interestingly, the lifespan of *miR-980* mutants at 25°C was similar to control and only at 33°C did they start dying significantly earlier than *wt* animals (50% lethality noted at 14.3 days vs. 21.0 days respectively) (Table 2, Figure 6d). Based on these results we hypothesize that the reduction of miR-980 under high temperature stress makes flies immobile, which is to some extent a protective mechanism that can at least preserve muscle tissue for a period of time. However, based on the lifespan studies, it appears that other adverse effects can be a result of this yet undefined mechanism. This is particularly interesting since we did not detect miR-980 expression in muscle, but in the nervous system (Figure 5d). These data suggest the miR-980 has a specific function in the nervous system that influences muscle functioning and subsequently longevity under hyperthermic stress.

### miR-252 is required for proper muscle development

We took advantage of the null *miR-252* mutant to examine its possible functionality. We initially noted that young three to four day old animals already had degenerated muscles relative to the *wt* animals (18.0% vs. 4.1% respectively, Table 3, Figure 6e,f). We additionally aged and applied hyperthermic stress to control and *miR-252* mutant animals and again assayed muscle degeneration. Though *miR-252* mutants continued to have more muscle degeneration than *wt* animals, the relative amount of muscle degeneration was not amplified as a result of aging or stress (Figure 6e).

**Table 3:** **Muscular dystrophy-related phenotypes in *miR-252 *mutants**

**Genotype**	**Longevity**		**Temperature-sensitive mobility**	**Muscle degeneration**		
	***50% survival (day)***		***Relative time of 50% mobility***	***3-4 days***	***18 days***	***6 days***
	**25°C**	**33°C**	**38±1°C**	**25°C**		**33°C**
*Control*	38.1±7.5	19.3±2.7	1.00±0.22	4.13 %	4.73 %	6.59 %
	(n=103)	(n=137)	(n=74)	(n=484)	(n=528)	(n=167)
*miR-252*	30.2±2.2	18.3±1.7	0.56±0.11	18.04 %	11.67 %	17.87 %
	(n=101)	(n=143)	(n=78)	(n=632)	(n=240)	(n=621)

Contrary to *miR-980* mutants, *miR-252* mutants had significant difficulties remaining mobile at the high temperature (Table 2, Figure 6g). As we only were able to confirm expression of miR-252 in the nervous system (Figure 5f), this phenotype could be indicative of improper neuron-muscle communication. Additional lifespan testing did not reveal that loss of miR-252 caused accelerated mortality (Figure 6h). Future experiments are aimed at a more in depth analysis of the role of miR-252 and its relationship with the DGC.

## Discussion

Under stressful conditions an organism can respond by adjusting metabolism, an important survival mechanism to address new cellular needs dependent upon the condition. When stress is applied, it is not logical for a cell to undergo a large transcriptional reorganization and generate new cellular proteins. It would be much easier to have a quicker response in place that can be mediated via miRNAs. Indeed it has been reported that metabolic rate reduction is linked to miRNA expression [43].

We have reported in the past that *Dg* mutants do not metabolically respond correctly to a low energy diet, where their metabolism remains high compared to *wt* animals [18]. We have additionally seen that *Dys* and *Dg* mutants, respectively, have elevated and decreased cellular levels of reactive oxygen species in animals housed at 29°C [23]. We have further observed temperature-related trends relative to CO_2_ output and ATP levels (unpublished data). Additionally, *Dg* has been implicated to be involved in starvation response by interacting with the mitochondrial ribosomal protein mRpL34 to regulate epithelial cell polarity [44]. The totality of these data suggests that the DGC and, particularly, Dg is involved in cellular homeostasis and stress response in general.

Here we were able to determine that in the absence of Dys or Dg the levels of miR-124 and miR-iab-4-5p fail to undergo the appropriate downregulation seen in *wt* animals in response to high temperature stress. It will be interesting in the future to determine if this lack of proper response contributes to the rapid progression of muscle degeneration that occurs when mutants are stressed. miR-124 has been shown to be specific to the nervous system and can be regulated by fragile X mental retardation protein 1 (dFR1) [45]. miR-iab-4-5p inhibits the homeodomain protein Ultrabithorax, and when ectopically expressed causes a homeotic transformation of halters into wings [46]. Additionally, we have revealed in our screen that miR-959 is a general stress response miRNA that is significantly downregulated in the hyperthermic condition regardless of the genomic background. Due to the DGC being implicated in a general stress response, not just hyperthermia, it will be of great interest to determine if these are general stress response miRNAs as well.

Prior studies on how muscle diseases affect the miRNA profile have been limited to muscles from mammalian disease models or biopsies from patients. Such studies have revealed in these tissues that misregulated miRNAs are involved in multiple biochemical pathways [13]. In agreement, transcriptomic analysis shows dysregulated genes associated with not only muscle cell contraction, but ion channels, metabolic pathways, and kinases in dystrophin deficient myotubes [47]. A more detailed analysis in muscle revealed a signaling pathway involving Dys-Syn-nNOS regulated histone modification allowing for proper transcription of miRNA genes [12]. We have expanded on these previous works here and found that loss of Dg, similarly to Dys loss, alters the miRNA profile. Furthermore, a signaling pathway involving Dg-Dys-Syn1 exists and can also regulate at least three miRNAs.

Dg-Dys-Syn1 regulates expression of miRNAs with different potential targets implemented in multiple developmental processes. Accordingly, the DGC is present in multiple tissues postulating that this complex has a more subtle housekeeping function. The Dg-Dys-Syn1 regulated miR-980 is likely to be primarily involved in nervous system development tuning axon guidance. Putative targets involved include transcriptional regulators in well-conserved pathways including TGF-β and Epiderman Growth Factor (EGF) signaling. The downregulation of miR-980 in pupae relative to the expression levels at other life stages supports that miR-980 is ‘off’ to allow for higher levels of proteins necessary for metamorphic neurological remodeling.

miR-956 is likely to be necessary to keep muscle development genes at low levels when no longer needed. The small number of potential target genes that is necessary for proper muscle development include the TGF-β ligand *glass bottom boat* (*gbb*)*,* the transcription factor *erect wing* (*ewg*), the translation repressor *pumilio (pum*) and *Ecdysone receptor (EcR*), a gene well known to be involved in temporal regulation of growth and development [48,49]. Based on our expression analysis miR-956 is ‘on’ in adult stages in the thorax and abdomen where it can potentially guard the expression of these genes so that muscle cells remain quiescent. Interestingly, very little expression was found in the ovaries even though there are muscle sheaths around each ovary and oviduct, and a muscle sheath connects the two ovaries to the uterus. This suggests that miR-956 is not expressed at the same level in all muscle types, and that striated flight muscles and those of the body wall lining the cuticle of the abdomen have a different miRNA profile than smooth muscle tissues. Dystrophin is considered a terminal differentiation gene in mammalian muscle cells, but no correlative studies have been conducted in *Drosophila*. Our data showing that Dys controls the levels of a potential muscle cell terminal differentiation miRNA in *Drosophila* suggests that Dys does have a similar role in this system.

In *Drosophila*, Syn1 and Syn2 have not been validated as components of the DGC, but our results in this study support an association between Dg, Dys and Syn1. The embryonic expression of *Drosophila* DGC proteins found that *Syn1* is expressed in the brain and the ventral nerve cord, while *Syn2* is expressed in mesoderm-derived tissues [16]. Interestingly, all three miRNAs studied here can be regulated by the Dg-Dys-Syn1 pathway. miR-956, miR-980 and miR-252 were found to be present in the nervous system and miR-956 is also present in the musculature. We clearly see a reduction in miR-956 levels in *tub-Gal4/dsSyn1* animals, suggesting that, at least in adult animals, Syn1 signaling could be present in the mesoderm as well. Accordingly, a study examining both Syntrophins in *Drosophila* reported that Syn1 and Syn2 can partially compensate for each other’s functions indicating that they can be expressed in the same tissues [50]. It is possible that the regulation of miRNA levels by the Dg-Dys-Syn1 pathway seen in our study is linked to neuronal Nitric Oxide Synthase (nNOS) signaling, which was reported in mammalian muscle [12]. Here we have shown that miR-956, miR-980 and miR-252 are all downregulated in mutants with *RNAi* against *Syn1* indicating that a comparable pathway is present in *Drosophila* that is not only present in muscle, but other tissues as well (i.e. the nervous system).

NO (Nitric Oxide) is a common regulatory molecule that has been shown to be involved in numerous cellular processes including metabolism of energy substrates, diversion of oxygen to nonrespiratory substrates, generation of reactive oxygen species, neurotransmission, and apoptosis to name just a few [51-53]. In *Drosophila* there is only one NOS gene (*dNOS*) that is expressed in imaginal discs during development, and injection of NOS inhibitors prior to metamorphosis results in major developmental defects [54]. This is interesting because based on the gene ontology of many of the putative targets, miR-252 is involved in regulation of imaginal disc morphogenesis. In adult animals, the *dNOS* transcript is found preferentially in heads, but not bodies [55], and the primary adult tissue that we see miR-252 and miR-980 expressed in is the adult head. Future efforts will involve determining if the regulation of these miRNAs is dependent on NO signaling in *Drosophila*. Despite extremely low levels of miR-252 expression, *miR-252* mutants do not show any gross morphological defects. However, its large number of targets involved in several processes allow us to speculate that it is a general housekeeping miRNA that fine tunes processes that have been first controlled via other mechanisms.

The mechanism of miRNA regulation by Dys and/or Dg is not fully known at the current time. Even though a pathway has been elucidated involving syntrophin mediated signaling for some miRNAs, the misregulation of others is mechanistically elusive. We have additionally seen in this study that Dg levels impact those of Dys and both affect the levels of Syn1. This is in accord with another study that found DGC expression profiles are interrelated [56]. One explanation of how DGC components regulate other complex proteins is through miRNA regulated silencing. Accordingly, two prior studies in vertebrates have shown that Dys and β1-syntrophin can be targeted by miRNAs [57,58]. Another intriguing fact is that DGC components have been found in the nucleus, where one Dys isoform was found in the nucleus of HeLa cells and C_2_C_12_ muscle cells in a complex with β-dystroglycan, β-dystrobrevin and nNOS [59,60]. Perhaps in the near future it will be determined if dystrophin and its associated proteins can directly regulate transcription of downstream genes.

In order to put into context the limitations of our study we would like to point out that here one sample per genotype per condition was analyzed via microarray. The sample consisted of 3–5 whole adult male animals. By combining multiple animals into one sample we hoped to minimize random variability in miRNA expression levels in any single animal. This random variability should have been normalized as ‘background noise’. Due to the large number of samples we were interested in for this study (3 genotypes X 2 conditions) we did not perform replicate microarray experiments. Additionally, due to this experimental design we set a cutoff value for differential expression at 0.5 and 1.5 that of the control for down and upregulation respectively. Therefore, it was very important to verify the microarray data we obtained via another method. Here we chose RT q-PCR analysis of fifteen miRNAs using pooled samples and performed three to five biological replicates. Of the 135 comparisons made using the RT q-PCR data, 79 (58.5%) agreed with the microarray data. Though this could be due to caveats of our experimental design, it has been reported that upon comparison of RT q-PCR based arrays vs. microarrays like the one used here, the former has superior sensitivity and specificity [26]. An additional limitation of our study is that we used whole animals which unfortunately does not allow determination of the tissue where specific miRNAs are misregulated.

## Conclusions

This study shows that in *Drosophila* similar to humans, loss of Dys alters the miRNA profile in a manner that is not exclusive to a signaling pathway involving Dg and Syn1. In addition to information obtained by previous studies, we have provided evidence in an *in vivo* animal model that hyperthermic stress can alter miRNA levels; a process that is defective in dystrophic mutants. This work supports that the DGC is involved not only in muscle maintenance, but in general for proper development of the whole organism. We also introduce the idea that the DGC has the capability of conducting necessary cellular pathways. These data suggest that one explanation for stress-induced dystrophy in *wt* animals and stress induced hyperdystrophic symptoms in DGC mutants can be miRNA response related. Our study adds to the growing knowledge of a strong link between the DGC and nervous system differentiation and functionality.

## Methods

### Fly Strains and Genetics

Fly stocks were maintained at 25°C on a standard cornmeal-agar diet unless otherwise stated. Fly strains used in this study are loss or function mutants *Dg*^*O86*^*/CyO*, *Dg*^*O55*^*/CyO* (both *Dg* alleles are point mutation induced resulting in no functional Dg protein [61]) and *DysDf* (deletion of the *Dys* gene generated by outcrossing the deficiency *Df(3R)Exel6184*[61]). The *DysDf* allele is homozygous viable and also removes five unnamed genes believed to be involved in general substrate transport, five with no known function and a sno-RNA gene with no known function. Despite this, the allele removes all isoforms of Dys meaning that we can use it to detect miRNAs that are altered in tissues where all Dys isoforms may be differentially expressed since long and short isoforms have been shown to have functions in the nervous system and muscles respectively [17,62]. The wild type strains *w*^*1118*^, *Oregon-R-C* (referred to as *Oregon R*) and *tub-Gal4/TM3* were obtained from Bloomington *Drosophila* Stock Center. *P[GawB]CG3777*^*NP3544*^ (P-element insert in the *miR-980* gene resulting in a 70% reduction of expression, *miR-980* hypomorph (Additional file 1), stock #113334) and *PBac[SAstopDsRed]LL04028* (PiggyBac element inserted in the *miR-252* gene resulting in no detectable expression, *miR-252* amorph (Additional file 1), stock #140830) were obtained from the Kyoto *Drosophila* Genetic Resource Center. The RNAi line targeting *Syntrophin-like 1* (*dsSyn1*, stock # 7152R-2) was obtained from the National Institute of Genetics, Japan.

### microRNA microarray

To prepare flies for microarray screening, when flies were one day old they were either left at 25°C for three days or shifted to the stressful temperature of 33°C for three days. Males were then collected and RNA was extracted from 3–5 flies per genotype per condition. Total RNA for microRNA microarray analysis was extracted using Trizol (Invitrogen) following the manufacturer’s protocol from adult 3–5 day old male flies. RNA quantity and purity were assessed by electrophoresis on an ethidium bromide stained agarose gel and a Nanodrop ND-1000 spectrophotometer measuring the A260/A280 ratio. The RNA was sent to the BioCat company for microarray analysis where they used the GenoExplorer^TM^ Biochip platform. Gene nomenclature is based on miRNAs registered and annotated in miRBase (release 16) at the Wellcome Trust Sanger miRNA registry. The GeneExplorer labeling system uses a biotin labeling system. Multiple positive and negative controls were included on the chip where positive controls were *U6-296*, *U6-337*, *5S rRNA*, and *tRNA-M2* which also served as loading controls. Negative controls were used for chip background. Each probe is printed in triplicate on the chip. In order for a signal to be considered real it must have exceeded the threshold set by the average signal from the combined negative controls plus 2X their standard deviation. Signals were then normalized to the average of those from the positive controls. The fold change of each individual probe was then determined by normalizing the questioned genotype to that of the control genotype. Only values that had a value below 0.5 or above 1.5 were deemed to have a significantly different level of expression. If a signal in the control sample was 0, then a signal of ≥ 0.2 by the test sample was considered to be increased. Inversely, if a signal in the control sample was ≥ 0.2 and the test sample was 0 then it was considered to be decreased. These thresholds were set to capture as much data as possible with this initial testing. Stress and dystrophy could cause subtle changes in miRNA levels that would cause subtle changes in gene regulation. However, over the shortened lifetime of these animals it is suspected that these minor changes can influence and add to the detrimental physiology that is characteristic of muscular dystrophies. Heatmaps and clustering of array data was done using the R program version 2.12.2.

### Quantitative reverse transcription PCR (RT-qPCR)

Total RNA was extracted using Trizol reagent (Invitrogen) following the manufacturer’s protocol. To determine miRNA levels, TaqMan microRNA assays (Applied Biosystems) were used where 2S rRNA was used as an endogenous control. Reverse transcription was done on a T100 thermocycler (Bio-Rad) and amplification was done with a StepOne Plus thermocycler (Applied Biosystems). Sequences used to design the TaqMan microRNA assays were obtained from FlyBase release 5.42. To determine mRNA levels, RNA was reverse transcribed using the High Capacity cDNA Kit following the manufacturer’s protocol (Applied Biosystems) and qPCR was carried out using the Sybr Green master mix (Applied Biosystems). For mRNA, Ribosomal Protein L32 (RpL32) was used as an endogenous control. Primers used were as follows: Dg forward - ACT CAA GGA CGA GAA GCC GC, Dg reverse - ATG GTG GTG GCA CAT AAT CG, Dys forward - GTT GCA GAC ACT GAC CGA CG, Dys reverse - CGA GGG CTC TAT GTT GGA GC, Syn1 forward – GGCATTGAACCAGACGAGGG, Syn1 reverse – AATCTCAAATACATCGACCC, robo forward – ACTGGAAGAGGTACCAATGAT, robo reverse – AGGAATTGGGTCACTTAAGTG, vimar forward – CACAAAGTAGATGCAGTGGC, vimar reverse - TCGCCTGCGAACTCATCGTAT, RpL32 forward – AAGATGACCATCCGCCCAGC and RpL32 reverse – GTCGATACCCTTGGGCTTGC. All reactions were run at least in triplicate with appropriate blank controls. The threshold cycle (CT) is defined as the fractional cycle number at which the fluorescence passes a fixed threshold. The ΔC_T_ value is determined by subtracting the average RpL32 or 2S rRNA C_T_ value from the average tested C_T_ value. The ΔΔC_T_ value is calculated by subtracting the average ΔC_T_ of the control sample from the ΔC_T_ of the suspect sample. The relative amount of mRNA or miRNA is then determined using the expression 2^-ΔΔCT^. All raw PCR data are in Additional file 1.

The one-tailed Student’s t-test was used to determine statistical significance of the relative amount of miRNA or mRNA. Presented error bars represent the standard deviation from the mean.

For determination of miRNA expression levels at different developmental time points and in tissues of the adult fly, embryos were collected ~20 hrs after eggs were laid, larvae were collected in the mid to late 3^rd^ instar stage, pupae were collected ~3 days after pupation and adult males were collected 3–5 days after eclosion. Separate body parts were taken from 3–5 day old male flies with the exception that ovaries were dissected from females.

### In situ hybridization

miRCURY LNA probes were purchased from Exiqon that are complementary to *Drosophila* miR-956 (product # 21431–15), miR-980 (product # 21455–15) and miR-252 (product # 21423–15). Tissues were dissected, fixed in 4% formaldehyde in PBS, dehydrated and stored at −20°C overnight or longer. Tissues were then rehydrated, treated for 10 min (muscles) or 1 hr (brains) with Proteinase K solution (50 μg/ml Proteinase K in 50 mM Tris–HCl pH 7.5, 50 mM EDTA), and post-fixed in 4% formaldehyde in PBT for 30 min. Tissues were prehybridized for 1 hr in hybridization buffer (50% formamide, 25% 20X SSC, 5 mg/ml Torula yeast RNA (Sigma), 0.1% Tween 20). Hybridization was carried out overnight at 60°C with a 40 nM probe concentration in hybridization buffer. Post hybridization washing was done at 62°C with three subsequent 20 min washes with hybridization wash solution (no yeast RNA), 50/50 v/v hybridization wash solution/PBT, PBT. Tissues were then blocked for 1 hr in Western Block (Sigma). Anti-DIG was diluted 1:2000 in block and incubated with tissues overnight at 4°C. Colorimetric detection was done with 10 μl/ml NBT (Roche) in staining buffer (0.05 M Tris pH 9.5, 0.05 M MgCl_2_, 0.1 M NaCl, 0.1% Tween 20) for approximately 30–45 min. Analysis was done using a Zeiss LSM 700 microscope.

### Gene ontology of predicted miRNA targets

Conserved *Drosophila* targets were determined for miR-252, miR-956 and miR-980 using targetscan.org (Release 6.1) [32] and microRNA.org (August 2012 release) [33]. The two lists from each database were compared per miRNA and only those targets that agreed among the databases were considered further. The Generic Gene Ontology (GO) Term Finder tool hosted by the Lewis-Sigler Institute for Integrative Genomics, Princeton University [34] was used to find GO process terms related to each group of miRNA targets with a p-value cutoff of 0.01. Visualization was done with the help of Revigo software [63] with allowed similarity equal to 0.5.

### Histology

To prepare *Drosophila* tissue sections, flies were immobilized in collars in the required orientation and fixed in Carnoy fixative solution (6:3:1, Ethanol:Chloroform:Acetic acid) at 4°C overnight. Tissue dehydration and embedding in paraffin was performed as described previously [64]. 10 μm histological sections were prepared using a Hyrax M25 (Zeiss) microtome and stained with hematoxylin and eosin. All chemicals for these procedures were obtained from Sigma Aldrich. Muscle analysis was done using a light microscope (Zeiss). The amount of muscle degeneration was calculated by counting the number of intact and degenerated indirect flight muscles (up to 12/animal) where the data is presented as a percentage. The two-tailed Chi-square statistic was used to assay for significance. Between 175 and 350 muscles were counted/genotype.

### Elevated temperature mobility assay

This test was adapted from one previously described [64]. 3–5 day old flies were placed in a preheated vial kept at ~38°C and scored every thirty seconds for flies that were still able to move. At least five trials were conducted per genotype. The time point when 50% of the flies were immobile is reported and a two-tailed Student’s t-test was used to test for statistical significance.

### Longevity

Immediately after eclosion adult males were kept on a standard diet at 25°C or 33°C and scored every 2–4 days for longevity rate. Experiments were done in at least triplicate with thirty flies in each trial per genotype tested. Statistics were conducted using the two-tailed Student’s t-test.

## Competing interests

The authors declare that they have no competing interests.

## Authors’ contributions

AKM and HRS conceived of and designed the study. AKM, EVE, NNH and MMK conducted experiments and AKM, EVE and HRS interpreted the data. AKM and HRS wrote the manuscript. All authors read and approved the final manuscript.

## Supplementary Material

Additional file 1 Experimental Data. Click here for file
